# PBK/TOPK: An Effective Drug Target with Diverse Therapeutic Potential

**DOI:** 10.3390/cancers13092232

**Published:** 2021-05-06

**Authors:** Hai Huang, Mee-Hyun Lee, Kangdong Liu, Zigang Dong, Zeayoung Ryoo, Myoung Ok Kim

**Affiliations:** 1 Department of Animal Science and Biotechnology, ITRD, Kyungpook National University, Sangju 37224, Korea; huanghai@knu.ac.kr; 2 China-US (Henan) Hormel Cancer Institute, Zhengzhou 450008, China; kdliu@zzu.edu.cn (K.L.); dongzg@zzu.edu.cn (Z.D.); 3 College of Korean Medicine, Dongshin University, Naju, Jeollanamdo 58245, Korea; mhlee@dsu.ac.kr; 4 Department of Pathophysiology, School of Basic Medical Sciences, The Academy of Medical Science, College of Medical, Zhengzhou University, Zhengzhou 450001, China; 5 School of Life Science, Kyungpook National University, Daegu 41566, Korea

**Keywords:** TOPK, cancer therapy, signaling pathway, inhibitors

## Abstract

**Simple Summary:**

Cancer is a major public health problem worldwide, and addressing its morbidity, mortality, and prevalence is the first step towards appropriate control measures. Over the past several decades, many pharmacologists have worked to identify anti-cancer targets and drug development strategies. Within this timeframe, many natural compounds have been developed to inhibit cancer growth by targeting kinases, such as AKT, AURKA, and TOPK. Kinase assays and computer modeling are considered to be effective and powerful tools for target screening, as they can predict physical interactions between small molecules and their bio-molecular targets. In the present review, we summarize the inhibitors and compounds that target TOPK and describe its role in cancer progression. The extensive body of research that has investigated the contribution of TOPK to cancer suggests that it may be a promising target for cancer therapy.

**Abstract:**

T-lymphokine-activated killer cell-originated protein kinase (TOPK, also known as PDZ-binding kinase or PBK) plays a crucial role in cell cycle regulation and mitotic progression. Abnormal overexpression or activation of TOPK has been observed in many cancers, including colorectal cancer, triple-negative breast cancer, and melanoma, and it is associated with increased development, dissemination, and poor clinical outcomes and prognosis in cancer. Moreover, TOPK phosphorylates p38, JNK, ERK, and AKT, which are involved in many cellular functions, and participates in the activation of multiple signaling pathways related to MAPK, PI3K/PTEN/AKT, and NOTCH1; thus, the direct or indirect interactions of TOPK make it a highly attractive yet elusive target for cancer therapy. Small molecule inhibitors targeting TOPK have shown great therapeutic potential in the treatment of cancer both in vitro and in vivo, even in combination with chemotherapy or radiotherapy. Therefore, targeting TOPK could be an important approach for cancer prevention and therapy. Thus, the purpose of the present review was to consider and analyze the role of TOPK as a drug target in cancer therapy and describe the recent findings related to its role in tumor development. Moreover, this review provides an overview of the current progress in the discovery and development of TOPK inhibitors, considering future clinical applications.

## 1. Introduction

T-lymphokine-activated killer cell-originated protein kinase (TOPK), also known as PDZ-binding kinase (PBK), is a serine–threonine kinase of the mitogen-activated protein kinase kinase (MAPKK) family and an oncogenic protein that regulates cell survival, proliferation, growth, apoptosis, and inflammation [1,2,3]. TOPK is also a mitosis kinase that is activated by the CDK1/cyclin B1 complex to promote cytokinesis through phosphorylation of polycomb repressive complex 1 (PRC1) [4,5] or anaphase-promoting complex (APC) ubiquitin ligase [6]; inactivation of protein phosphatase 1 alpha (PP1a) leads to phosphorylation at Thr9, which promotes TOPK activation [7]. Activated TOPK leads to chromatin condensation by facilitating phosphorylation of histone H3 at Ser10 in M phase; furthermore, the depletion of TOPK induced by siRNA causes cytokinetic defects in cancer cells [7,8]. Thus, TOPK functions in cancer development. In addition, TOPK has also been recognized as a metastasis-promoting kinase in cancer metastasis [9]. In addition, high expression of TOPK is correlated with oncogenic KRAS and BRAF mutations. Moreover, TOPK could mediate pathway activation independently of B-Raf or C-Raf, which indicates that a TOPK/extracellular signal-regulated kinase 2 (ERK2) feedback loop might bypass the negative feedback loop regulating the Raf/MEK/ERK pathway, which in turn promotes transformation potential. All of those based on ERKs are unique substrates for MEK1 or MEK2, and MEK proteins are downstream targets of Raf kinases [10]. Moreover, TOPK dysregulation can also potentiate cancer development and dissemination. Therefore, given that TOPK is highly transactivated in various types of cancer and related to aggressive tumor phenotypes [11,12,13], it is now considered a promising molecular target for the treatment of malignant tumors.

In terms of its mechanism, overactivation of TOPK promotes cancer cell proliferation, inflammation, and metastasis through activation of downstream signaling cascades, such as the MAPKs and ribosomal S-6 kinase (RSK), as well as transcription factors, including activator protein-1 (AP-1) and NF-κB [14,15,16,17]. Furthermore, TOPK expression is associated with H-Ras-induced cell transformation, activation of c-jun-NH2-kinase (JNK), and p53 expression via UVA, UVB, and DNA damage [18,19], respectively. In addition, TOPK promotes cell migration through regulation of the PI3K/PTEN/AKT-dependent or TGF-beta1/Smad signaling pathways [9,20]. Thus, TOPK performs an oncogenic cellular function, and its inhibition should be effective in cancer therapy. Furthermore, TOPK is a highly ranked radio-sensitizing gene, which further indicates its potential as a target to widen the therapeutic window given its differential expression between cancer and normal tissues [8]. Notably, however, TOPK expression is hardly detectable in normal tissues, except in the testis and fetal tissues. In addition, a TOPK inhibitor is not currently in clinical use. Even the effects of pantoprazole, an FDA-approved inhibitor that targets TOPK, have been reported in a colon cancer only [21]. Subsequent reports of HI-TOPK-032 and OTS964 TOPK inhibitors have indicated disadvantages in terms of solubility and toxicity, which limits the pace of clinical application [22,23]. In summary, TOPK is a novel oncogene that plays a crucial role in the occurrence and development of cancer. Targeting TOPK may help provide new therapeutics for cancer patients; therefore, it is an attractive potential target for the development of chemotherapeutic inhibitors.

## 2. TOPK Expressed Highly in Cancers and Is a Prognostic Marker of Cancer

**Figure 1:**
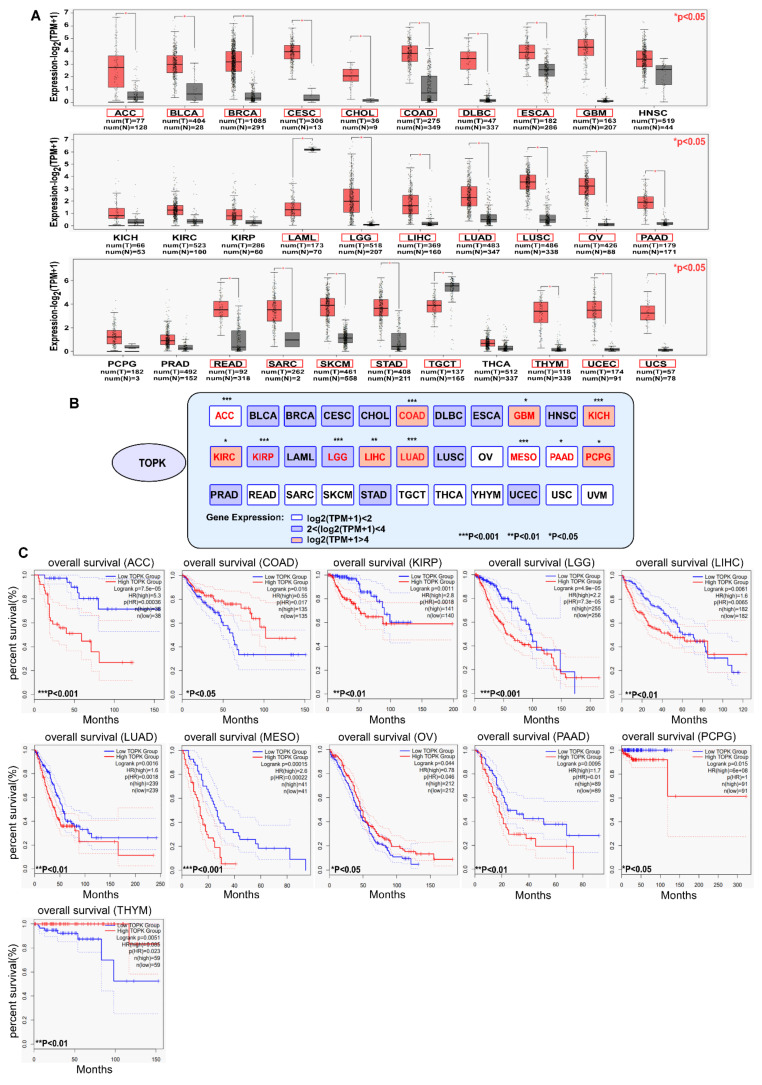
The expression of TOPK in cancers and TOPK expression related to overall survival of patients with cancers. (**A**) Comparison of the expression of TOPK kinases between tumor and normal tissues. The images and significance are from The Cancer Genome Atlas database. * *p* < 0.05, ** *p* < 0.01, and *** *p* < 0.001. (**B**) Correlation between TOPK kinase expression and patient overall survival. Red text: gene expression had significant relation with survival; black text: gene expression had no significant relation with survival. The survival data are derived from ULCAN database (http://ualcan.path.uab.edu/) (accessed on 25 March 2021). Samples were categorized into two groups for analysis: high TOPK expression (with TPM values above upper quartile); low/medium TOPK expression (with TPM values below upper quartile). * *p* < 0.05, ** *p* < 0.01, *** *p* < 0.001. (**C**) The correlation of TOPK expression in 11 types of cancer with clinical outcomes; the other 20 types of cancer showed no significant differences with overall survival (data not shown).

The expression of TOPK is significantly higher in various cancers compared with in normal tissues according to The Cancer Genome Atlas database (http://gepia.cancer-pku.cn/) (accessed on 25 March 2021), as shown in Figure 1, with exceptions for head and neck squamous cell carcinoma (HNSC), kidney chromophobe carcinoma (KICH), kidney renal clear cell carcinoma (KIRC), kidney renal clear cell carcinoma (KIRP), pheochromocytoma and paraganglioma (PCPG), prostate adenocarcinoma (PRAD), and thyroid carcinoma (THCA). Notably, TOPK expression is reduced in acute myeloid leukemia (LAML) and testicular germ cell tumors (TGCT) versus normal tissues. Indeed, samples from patients with 24 of 31 (Figure 1A, red pane, * *p* < 0.05) tumor types had markedly higher expression of TOPK, which further highlights the suitability of TOPK as a target for cancer treatment. By examining the relationship between survival and expression of TOPK (survival data are collected in Figure 1), it becomes clear that high levels of TOPK expression are significantly associated with survival in 11 of 33 tumor samples. Interestingly, bladder urothelial carcinoma (BLCA), breast invasive carcinoma (BRCA), cervical squamous cell carcinoma (CESC), cholangiocarcinoma (CHOL), esophageal carcinoma (ESCA), glioblastoma multiforme (GBM), HNSC, PRAD, stomach adenocarcinoma (STAD), and uterine corpus endometrial carcinoma (UCEC), all of which show TOPK upregulation, are not correlated with survival.

As an oncogene that is overactivated, leading to cancer development at a rapid rate, TOPK overexpression is a common molecular characteristic of human malignancies, and it has been reported in many types of human cancers, including colon, lung, esophageal, prostate, and ovarian cancers [9,11,12,13,24]. High expression of TOPK has also been linked to tumor aggressiveness, invasion, and metastatic spread in various malignancies. Therefore, TOPK expression may be associated with poor prognosis in these cancers under certain circumstances or considered a prognostic marker. Previous reports have shown that a significant relationship exists between TOPK expression and poor prognosis in various cancers, such as prostate, liver, lung, colorectal, and brain cancers [18,22,25,26]. High levels of TOPK expression are significantly associated with poor progression-free survival and overall survival in the early-stage cases of epithelial ovarian cancer [24]. In addition, patients with high TOPK expression levels and non-small cell lung cancer also show poorer overall and recurrence survival [9,27] when compared with patients with low TOPK expression. In clinicopathological patients with oral cancer, TOPK is significantly associated with prognosis in various models [28]. Furthermore, analysis of immunostaining for TOPK expression in cholangiocarcinoma from HCC has revealed that TOPK serves as predictor of survival in cholangiocarcinoma patients [29]. Similarly, immunohistochemical staining in colon cancer patients showed that TOPK expression is associated with clinicopathological features and mainly occurs in the cytoplasm and nucleus [30]. TOPK is also a novel prognostic biomarker and therapeutic target for chordoma; suppression of TOPK leads to significantly reduced chordoma cell proliferation and triggers an increase in apoptosis [31]. Koh et al. reported that TOPK is a biomarker for predicting outcomes in patients with primary central nervous system lymphoma [32]. PBK/TOPK is often overexpressed in gastric cancer; thus, it could be a crucial molecular marker to determine the malignant properties of gastric cancer cells and a target for molecular therapy in gastric cancer patients [33]. Moreover, application of fluorescently labeled TOPK inhibitor is used for cancer-specific imaging in a variety of tumors and can potentially improve patient care [34]; for example, the labeling of a TOPK inhibitor with F-18 has been exploited for positron emission tomography imaging in glioblastoma [35], and TOPK may represent a biomarker for malignant glioma [36]. Additionally, TOPK seems to be a valuable prognostic factor in patients with sporadic CRC with KRAS or BRAF mutations, as well as in patients with metastatic disease who respond to anti-EGFR therapies [37]. Hence, given its cancer-specific expression and known functions, TOPK is a potentially valuable cancer biomarker for patient stratification and risk assessment.

## 3. TOPK Promotes Proliferation

TOPK is a common cancer molecule in human malignancies, and several studies have revealed that it also plays crucial roles in cellular functions, including cell proliferation, DNA damage repair, the cell cycle, apoptosis, immune responses, and inflammation [7,15,18,30,38,39]. It is clear that TOPK is a potential therapeutic target in cancer. Below, we introduce the application of TOPK as a target in cancer therapy from various perspectives. 

The expression of TOPK is closely related to cell malignancy and the malignant potential of tumor cells. Previous reports have indicated that overexpression of TOPK promotes cell proliferation and tumor formation in JB6 epidermal cells both in vitro and in vivo [10,40,41]. In contrast, knockdown of TOPK in cancer cell lines suppresses tumor growth [10,42,43]. Moreover, the overexpression and oncogenic activities of TOPK are reported in cancer types. For example, TOPK expression is enhanced in many tumor cell lines compared with in nontransformed cells; its expression can be induced by IGF-I through the phosphatidylinositol 3-kinase (PI3K)/mTOR pathway and the ERK pathway [44]. It has been suggested that TOPK promotes cell growth and survival and is involved in the AKT and MAPK signaling pathways. Specific to cancers, TOPK is regulated by protein phosphatase 2A (PP2A) and BCR/ABL in leukemia and enhances cell proliferation, indicating that it may be a target of BCR/ABL [45], and has been associated with HTLV-1-transformed T-cell lines and ATLL-derived T-cell lines [17]. TOPK is a modulator of CEBPA activity in FLT3-ITD mutated AML cells; therefore, inhibition of TOPK reduces phosphorylation of CEBPA p42, which then becomes available to bind E2F1 and suppresses the transcriptional activity of E2F1/MYC [46]. Furthermore, suppression of TOPK is effective in inhibiting proliferation and survival in CSC subpopulations of nonsmall-cell lung cancer cells; this is thought to be related to suppression of FOXM1 activity, which plays a crucial role in the expression of late cell cycle control genes [40]. Moreover, given that TOPK interacts with the DNA binding domain (DBD) of tumor suppressor p53 and leads to a decrease in cell cycle regulatory proteins, such as p21, which consequently leads to cancer development and progression [18], PBK/TOPK may be an effective target for antineoplastic kinase inhibitors that induce apoptosis and suppress growth. Therefore, through targeting TOPK could suppress cancer proliferation.

## 4. TOPK Promotes Tumor Dissemination

TOPK putatively promotes metastasis; plays a role in epithelial–mesenchymal transition; and facilitates tumor invasiveness of lung, prostate, gastric, pancreatic, or breast cancer cells. Previous studies have suggested that TOPK/PBK promotes cell migration through regulation of the PI3K/PTEN/AKT pathway in lung cancer [9]. Jiang et al. (2020) showed that TOPK promotes metastasis via γ-catenin through Src/GSK3β/STAT3 signaling activated in esophageal squamous cell carcinoma. Through upregulation of TBX3 in TGF-β1/Smad signaling, TOPK also facilitates epithelial–mesenchymal transition and invasion of breast cancer cells [20]. In addition, TOPK has fundamental roles in the metastasis of other cancers, including colon cancer and melanoma, which are associated with the AKT, ERK, and β-catenin pathways [13,40]. Knocking down TOPK has been shown to decrease the migration of cancer cells both in vitro and in vivo [9,11,12]. Indeed, TOPK is highly expressed in tumor cells and facilitates metastasis; hence, targeting TOPK could suppress cancer metastasis. In addition, TOPK promotes tumor dissemination by direct phosphorylation of p53-related protein kinase (PRPK) at its Ser250 residue, which in turn regulates the phosphorylation status of p53 and AKT [47]. TOPK expression has also been related to distant metastasis of tumor cells. Studies have reported that TOPK expression in lung cancer is significantly higher than expression in paired paracancerous tissues and benign tumors, which represents an independent risk factor for lymph node metastasis and distant metastasis of lung cancer [43]. Furthermore, the metastatic activity of lung cancer cells (H1299) was increased by 2~3-fold after transfection with TOPK plasmid, whereas knockdown of TOPK inhibited metastasis [9]. Reports are increasingly suggesting that specific drugs can inhibit the activity of TOPK in an ATP-competitive—or non-competitive—manner to reduce cell proliferation and metastasis [2,48]. Thus, TOPK is related to the cancer metastasis.

## 5. TOPK Regulates the Cell Cycle

TOPK expression and activation levels directly regulate the cell cycle. Indeed, TOPK may be an important hallmark in this progress. TOPK affects the process of cell mitosis and promotes cell division by activating PRC1, which plays an important role in the formation of the spindle and its equatorial plan. Prior studies showed that TOPK could bind to PRC1 through its C-terminal glutamate aspartic acid repetitive sequence to promote the phosphorylation of PRC at the T481 site, which, therefore, increased the phosphorylation level of CDK1/cyclin B1 to PRC1 and eventually promoted cell cycle division [4]. In addition, some studies have reported that TOPK ubiquitinated by CHFR resulted in phosphorylated and inactivated PTEN, consequently inducing the activation of AKT, which is important for cell cycle progression during the G2 to M phase transition [49]. Studies have also revealed that TOPK expression levels and kinase activity are positively correlated with the number of cells at the G2/M phase in gastric carcinoma and prostate cancer, whereas silencing TOPK or inhibiting it activation can arrest the cell cycle in the G0 phase [50,51]. In this process, the cell cycle is arrested via TOPK and the CDK1/cyclin B complex formation, which prolongs the degradation of cyclin B. Meanwhile, the expression time of cyclin B during cell division is prolonged. However, Liu et al. [38] found that knockdown of TOPK resulted in G2/M cell cycle arrest in promyelocytes, a phenomenon that may result in hysteresis during the formation of the CDK1 and cyclin B complex. In another study, the activity of PP1a was inhibited after its phosphorylation (Thr320) by CDK1⁄cyclin B1 in early-to-mid mitosis [52]. Apparently, partial silencing of TOPK was unable to block cell cycle progression but altered the duration of the cell cycle phase. Thus, the TOPK expression pattern on the cell cycle correlated with that of cyclin B1. Furthermore, TOPK is also associated with the S phase in the cell cycle. An in vitro study has shown that TOPK plays a hitherto unknown role during S phase, suggesting that TOPK depletion promotes fork stalling and collapse under conditions of replication stress and exogenous DNA damage, thereby inducing cell apoptosis [53]. Moreover, activated TOPK phosphorylates histone H3 (Ser10) at the M phase, while the H3 histone variant CENP-A is an epigenetic marker critical for centromere identity and function. Yu et al. [54] reported that CDK1 and PP1α control the phosphorylation status of CENP-A Ser68, which orchestrates its cell-cycle-dependent deposition at centromeres. However, the relationship between TOPK and CENP-A at centromeres during the cell cycle remains to be fully explored. Prior studies showed that phosphorylation by PBK/TOPK was impaired when Thr450 was substituted to alanine (T450A) and that this lead to the isolation of homologous chromatids and the formation of F-actin polymerization in contraction rings [55]. Another study indicated that targeting the phosphorylated p97 site of TOPK could lead to mitosis discontinuity [7]. Moreover, TOPK-dependent transcriptional regulation of cyclin B2 was critical for tumorigenesis and radioresistance in GBM cells [56]. These findings suggest that TOPK is a critical target for cancer treatment in the inhibition of mitotic progress.

## 6. TOPK Induces Apoptosis Resistance in Tumor Cells

The abnormal expression of TOPK is not only associated with the cell cycle but also with apoptosis and its activation; thus, it confers resistance to drug-induced apoptosis and favors carcinogenesis. Previous reports have revealed that TOPK can downregulate the activation of p53 through its DBD. Furthermore, the expression of p53, its target gene, and cyclin-dependent kinase inhibitor p21 are upregulated in TOPK knockdown experiments [18]. Activation of TOPK in cancer may be promoted by inhibiting microRNA-mediated regulation [57]. In addition, restoration of miR-216b-3p expression in cancer cells is sufficient to inhibit proliferation, promote apoptotic cell death, and overcome TOPK-related downregulation of p53 and p21 [58]. Roh et al. [59] reported that PRPK is a novel kinase downstream of TOPK and a critical player in the promotion of skin carcinogenesis; they showed that knockdown of PRPK increased paclitaxel-induced apoptosis. However, the FDA-approved antibiotic cephalosporin [60] and synthetic compound ADA-07 [61] have been shown to suppress skin carcinogenesis by blocking TOPK activity. In addition, knockdown of TOPK inhibits Prx1 (Ser-32) phosphorylation and thereby induces apoptosis that benefits skin cancer therapy [62]; indeed, knockout or mutation of TOPK leads to the phosphorylation of Prx1 and significantly increases H2O2. The ability of cells to tolerate oxidative stress is ultimately decreased, and this triggers apoptosis [63]. Taken together, these findings indicate that targeting TOPK is efficacious for cancer drugs resistance and, therefore, induces cell apoptosis.

## 7. Others Factors: Inflammation, DNA Damage, and Autophagy

Little is known about the role played by TOPK in inflammation; however, TOPK is reported to be involved in ultraviolet (UV) light-mediated inflammation through phosphorylation of MKP1 and PRPK [3,47]. Furthermore, Seol et al. [15] found that in TOPK-depleted cells, LPS-induced inducible nitric oxide synthase (iNOS) expression was significantly diminished compared to that in control cells, whereas iNOS was induced by activation of TOPK through LPS/TLR4-induced signaling cascades, which suggests that TOPK is involved in LPS-mediated inflammation. This process can, therefore, result in cell migration and invasion in breast cancer [16]. On the other hand, LPS induces immune responses that elicit the production of NO; PGE2; and cytokines, such as TNF-α, IL-1β, and IL-6, in macrophages when its increases TOPK levels and phosphorylates serine or threonine residues in TOPK; this leads to the activation of HDAC1/HDAC2 that contributes to neuroprotective effects against cerebral ischemia reperfusion injury [64]. TOPK activation-mediated anti-inflammation is also suggested to be involved in remote limb ischemic postconditioning invoked protection against renal ischemia/reperfusion injury; thus, TOPK is a promising target for new drug development in the treatment of ischemic stroke.

In addition, PBK knockdown affects the DNA damage response of cells and enhances the sensitivity of cells to genotoxic agents. Previous studies have shown that TOPK acts as mediator of p38 growth-factor activation (which functions in motility), plays a part in the DNA damage sensing machinery, and contributes to γ-H2AX generation, whereas knockdown of TOPK can impair the generation of γ-H2AX [44]. Hence, PBK likely contributes to γ-H2AX generation, which is responsible for the recruitment of DNA damage response proteins to damage sites; however, its function in activation of DNA damage repair machinery may provide tumor cells with a more efficient repair response that would facilitate tumor growth. Furthermore, TOPK binds with and phosphorylates histone H2AX, which suppresses As3+-induced apoptosis in melanoma cells [65], and TOPK silencing reduces the number of γ-H2AX foci in MCF-7 cells following UV-induced DNA damage [66].

TOPK is related to MAPK, PI3K/AKT, mTOR, and other signal transduction pathways involved in autophagy regulation and, therefore, may participate in this process. Prior research has revealed that inhibiting autophagy increases paclitaxel/cisplatin-triggered apoptosis in cancer cells [67,68,69]. Lu et al. [70] reported that TOPK could bind with and phosphorylate ULK1 at the sites Ser469, Ser495, and Ser533, which led to the increased sensitivity of glioma cells to temozolomide by inhibiting autophagy. Ma et al. [71] found that ecotropic viral integration site-1 (EVI1) confers ovarian cancer cells with cisplatin resistance by inducing autophagy through targeting of the TOPK promoter region in ovarian cancer. In addition, microRNA-mediated autophagy regulation in cancer treatment also plays an important role in chemotherapy resistance/sensitivity [72]; for example, a previous report indicated that miR-216b enhanced chemosensitivity by regulating PBK [58]. These studies suggest that TOPK is a novel autophagy regulator and that targeting TOPK benefits the sensitivity of anticancer clinical drugs.

## 8. TOPK-Interacting Proteins Involved in Signaling Pathways

TOPK is a member of the novel MEK3/6-related MAPKK family that has crucial functions in various cellular processes. Although some studies have reported that ERK1/2 can only be selectively activated by MEK [73,74], Zhu et al. [10] found that ERK could also be activated by TOPK in colon cancer cells. Other studies have now indicated that knockdown of TOPK or treatment with TOPK inhibitors results in decreased ERK activity [1,15,22], while the expressions of c-MYC, NF-κB, and CREB, all of which are downstream of MEK, are also all reduced by such treatments. Furthermore, some typical kinases play crucial roles in many signal transduction pathways, and their activity is tightly regulated by two phosphorylation events [75,76]; thus, it can be seen that the autophosphorylation of proteins is also essential for regulating signaling pathways. Although the structural consequences of the autophosphorylation process of TOPK have not been reported, this role should be noted. TOPK can also activate other families of the MAPK pathway. For example, Dougherty et al. [77] found that TOPK also plays a crucial role in progenitors and is generally associated with the p38/MAPK pathway, indicating that TOPK is an important regulator of growth and self-renewal in progenitor. Furthermore, targeting TOPK could promote apoptosis by inducing ROS and activating the JNK/p38 signaling pathway [78]. Kim et al. [22] used a TOPK inhibitor to study colon cancer and found that phosphorylation of ERK–RSK was reduced by the inhibitor. The novel selective TOPK inhibitor SKLB-C05 has also been reported to block proliferation and metastasis of human CRC by regulating specific TOPK downstream signaling, including ERK1/2, p38, and JNK1/2/3 signaling [48]. Moreover, the activation of EGFR with the treatment of EGF in lung cancer cells was positively correlated with TOPK phosphorylation. Li et al. [79] found that TOPK was significantly upregulated and profoundly activated in lung cancer cells that exhibited resistance to EGFR-TKIs, which phosphorylated c-Jun, leading to an increased level of AP-1 [80]. It is suggested that TOPK is involved in MAPK constitutively activation. In addition, targeting the COX2/MET/TOPK signaling pathway blocks gefitinib resistance through AP-1 in lung cancer [81]. Therefore, TOPK is considered a MAPKK-like protein that is involved in ERK/MAPK, p38MAPK, and JNK signaling, possibly in a cell type-dependent manner.

**Figure 2:**
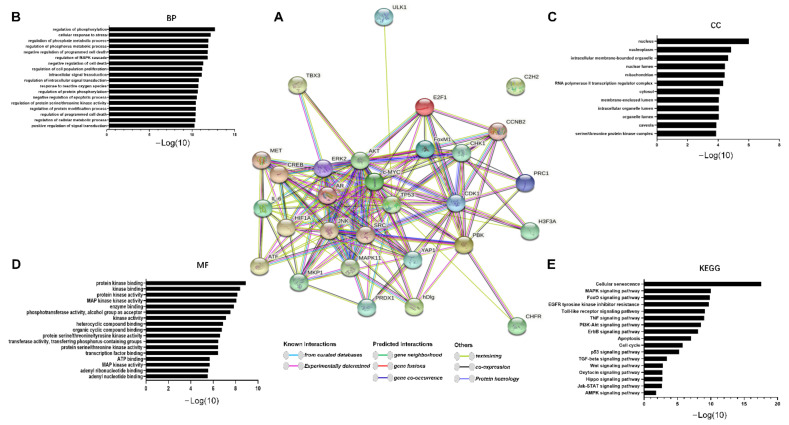
The TOPK interactome and functional enrichment of the network. (**A**) The interactome in the center was acquired through the STRING database based on the TOPK interaction proteins mentioned in Table 1. (**B**–**D**) Functional enrichment of network proteins that interact with TOPK. (**E**) Signaling pathway enrichment of network proteins that interact with TOPK.

TOPK is also involved in the PI3K/AKT pathway in tumor development. Recent results have shown that TOPK promotes cell migration by regulating the PI3K/PTEN/AKT pathway, whereas knockdown of TOPK has the reverse effect [9]. Previous studies also indicate that both the Ras-Raf-MEK-ERK and PI3K-AKT signaling pathways are constitutively activated through multiple mechanisms in cancers [14,82], suggesting that cross-talk and compensation exist between the two pathways. In addition, Jiang et al. [12] reported that TOPK promotes ESCC metastasis by activating the Src/GSK3β/STAT3 and ERK signaling pathways. Therefore, TOPK is an important signaling molecule in PI3K/AKT activation of ERK1/2 signaling. Based on these results, we determined the molecules activated and regulated by TOPK. In addition, we summarized TOPK-related proteins, including upstream, downstream, and transcription factors, as previously reported in the literature (Table 1). We then used the STRING database to obtain an interactome network based on TOPK-interacting proteins (i.e., those listed in Table 1; see Figure 2A). Furthermore, we used the Kyoto Encyclopedia of Genes and Genomes (KEGG) pathway and Gene Ontology (GO) enrichment analyses to further investigate these proteins. Our results indicated that TOPK-related proteins are involved in processes including mitosis and cell cycle progression and play important roles in kinase binding. These proteins are either directly or indirectly related to key molecules in crucial signaling pathways, such as the MAPK, FOXO, PI3K–Akt, ad TNF, and p53 pathways (Figure 2B–E).

## 9. Development of TOPK Inhibitors and Potential Compounds

Targeting therapy has become a popular trend in drug design. To date, HI-TOPK-032 [22] and OTS514/OTS964 [23] are three common inhibitors developed to specifically target TOPK (Table 2). HI-TOPK-032 belongs to the class of ATP-competitive inhibitors and can be a potent therapeutic agent, in vitro and in vivo, against cancers such as colon cancer, skin cancer, and nasopharyngeal carcinoma [22,78,93]. Either using HI-TOPK-32 as a TOPK inhibitor or knocking down TOPK inhibits the viability of cell growth and sphere formation in human brain tumor-derived glioblastoma stem cells [94], strongly supporting the use of TOPK as a therapeutic target in CSCs and the potential benefits for treating patients with CSC-enriched tumors.

OTS514 and its dimethylated derivative OTS964 were screened from a small molecular compound library and both have been developed into TOPK kinase inhibitors with high affinity and selectivity (IC50s: 1.5–14.0 nM for OTS514; 7.6–73.0 nM for OTS964). Furthermore, OTS514 has now been tested in a phase I trial against acute myelogenous leukemia. These two compounds have been shown to effectively inhibit the growth of different types of human cancer cells [95] and significantly suppress tumor growth in human lung/ovarian cancer in a mouse xenograft model. Interestingly, transplanted tumors were shown to completely subside following treatment with liposomes of OTS964, whether administered intravenously or orally in its free form. Moreover, OTS964 overcame the weight loss due to blood toxicity caused by OTS514. However, Sugimori et al. [96] found that surviving glioma stem cells restart growth as tumor spheres after being treated over a long period, which clearly restricts the application of OTS964 in cancer treatment. Furthermore, [18F] FE-OTS964 has been produced from OTS964; this preclinical drug specifically binds to TOPK, and test results have revealed that it reduces tumor uptake ratio from 3.06 ± 0.30% ID/cc to 1.40 ± 0.42% ID/cc, which was reduced in animals co-injected with an excess blocking dose of OTS541. These findings suggest that FE-OTS964 can be traced to TOPK more effectively.

ADA-07 is a novel, synthesized, ATP competition-type TOPK kinase inhibitor [61]. Currently, ADA-07 is mainly applied against skin cancer induced by UVA. It inhibits the phosphorylation of ERK1/2, p38, and JNKs by targeting TOPK, and then inhibits the activity of AP-1. Furthermore, FDA-approved proton pump inhibitors (PPI), such as ilaprazole, cephradine, and pantoprazole, have also been shown to target TOPK [21,97]. Meanwhile, PPIs have been used in anticancer drugs for the first time by targeting TOPK independent of gastric acid-related diseases. Given that they have shown favorable preclinical results in animal models in which they specifically targeted TOPK, they may soon be used to treat cancer patients. However, lansoprazole and rabeprazole showed no clear binding to TOPK, despite belonging to the group of proton pump inhibitors [97]. 

Recent reports have shown that the novel selective TOPK inhibitor SKLB-C05 could be effective against nine types of kinase activity and that a >10-fold disparity exists between the IC50 of TOPK and eight other kinases tested. Gao et al. also showed that inhibiting the activity of TOPK led to downregulation of ERK1/2, p38, and JNK1/2/3, as well as FAK/Src–MMP signaling, which further suppressed cell proliferation and migration [48].

Sulfasalazine, another FDA-approved drug, has also been identified as a TOPK inhibitor. Sulfasalazine has been shown to inhibit proliferation and metastasis of thyroid cancer by influencing the PI3K/AKT signaling pathway through TOPK [98]. Furthermore, sulfasalazine has already been approved for clinical use; however, its potential toxicity in thyroid cancer has yet to be tested.

Because the role of TOPK is becoming clearer and preliminary results are being achieved by targeting TOPK in cancer, discovering more inhibitors will become a challenge in the future. Natural compounds with multiple targets are already used in cancer therapy. For example, some phytochemicals derived from edible plant plants, including ginger, grapes, and tomatoes, have been reported to interfere with a specific stage of the carcinogenic process [99]. Below, we introduce some representative natural phytochemicals that could inhibit the activity of TOPK kinase directly (Table 2).

Acetylshikonin has been reported to inhibit cell proliferation in several cancers. Zhao et al. [1] confirmed that acetylshikonin inhibits the TOPK/ERK signaling pathway by directly targeting TOPK but not MEK; they also demonstrated the specificity of acetylshikonin binding with active TOPK in the presence of ATP. This compound was also found to suppress human leukemia cells and oral cancer proliferation through NF-κB signaling and AKT signaling, respectively [115,116]. In a separate study, Zhao et al. [2] found that 3-deoxysappanchalcone could reduce cell growth by directly targeting TOPK competitive ATP in colon cancer. In other research, Diao et al. [105] revealed that TOPK is a direct target of baicalin, a natural flavonoid glycoside extracted from the traditional Chinese medicine Baical skullcap root, which inhibits lung cancer cell growth through downregulation of histone H3 and ERK2 both in vivo and in vitro. Another compound, ginsenoside Rh2 (GRh2), is the main active chemical constituent of ginseng, the root of *Panax ginseng* Meyer. Prior studies have revealed that it is pharmacologically efficacious against cancers [117,118,119]. Yang et al. [108] found that GRh2 directly binds with TOPK and directly inhibits TOPK activity in HCT-116; in a mechanistic study, they demonstrated that GRh2 inhibits the phosphorylation levels of ERK1/2 and H3 in colon cancer cells. The same mechanism also applies to the compounds cyanidin-3-O-glucoside and eupafolin [41,107]. Interestingly, coffee is a rich source of dietary phenolic phytochemicals and inhibits colon cancer metastasis in mice as well as neoplastic cell transformation by suppressing ERK phosphorylation; these effects are associated with direct inhibition of MEK1 or TOPK activity [106]. Indeed, affecting the phosphorylation of histone H3 and ERK seems to be crucial for the prevention of cancer via TOPK targeting. Gossypetin is a hexahydroxylated flavonoid that exists in many flowers including hibiscus; Wang et al. [109] found that it could suppress solar UV-induced phosphorylation of TOPK, p38 MAPK, ERK1/2, and H2AX through direct inhibition of TOPK kinase. Notably, Kim et al. [120] found that gossypetin does not phosphorylate the related JNK or ERK MAPKs when p38 MAPK is inhibited in esophageal cancer, which further highlights how ERK is a direct substrate of TOPK. Paeonol is isolated from plants used in traditional Chinese herbal medicine such as Moutan cortex. Xue et al. [113] found that targeting TOPK with paeonol could reduce the phosphorylation of p38 or JNK MSK1 and histone H2AX in HaCat cells or JB6 Cl41 cells. Scutellarin (SCU) is an active ingredient extracted from *Erigeron breviscapus* (Vaniot) Hand.-Mazz that has been reported to directly bind with TOPK and inhibit TOPK activity in vitro. Moreover, SCU affects RPMI7951 cell growth by regulating the phosphorylation levels of ERK1/2 and histone H3 in a time- and dose-dependent manner [114]. Kim et al. [121] found that SCU was a potential AKT inhibitor that inhibits patient-derived xenograft ESCC tumor growth. Given that AKT can act as a substrate of TOPK, it would be worthwhile studying whether SCU affects the expression of AKT by targeting TOPK.

Taking all of these results into consideration, the targeting of TOPK clearly has therapeutic potential for the treatment of various human cancers. Given that natural compounds have high efficacy and low toxicity, they have become the focus of research aimed at discovering their pharmaceutical value in chemoprevention and chemotherapy. These compounds are currently an important part of cancer prevention and continue to provide a source for anticancer chemotherapeutic drug development. Therefore, targeted oncology drug research must continue to include natural products that can offer new opportunities for monotherapy or combination therapies against tumor cytotoxicity.

## 10. Discussion and Perspectives

Target therapy will play an important role in the future of cancer therapy, and inhibiting kinase phosphorylation to block pathway development is at the core of targeted drug development. Kinases can regulate a diversity of biological activities, including cell proliferation, metabolism, transcription, and differentiation; thus, they are considered promising drug targets for the treatment of various diseases ranging from inflammatory disease to cancers [122].

**Figure 3:**
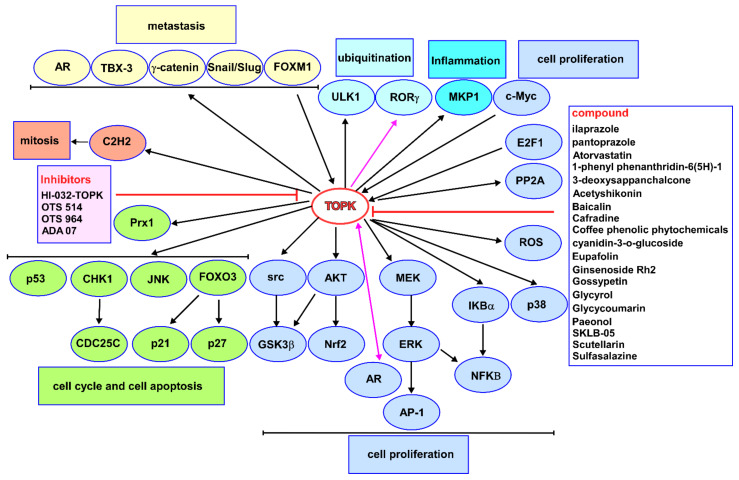
Schematic diagram of the biological roles involved in the proteins interacting with TOPK and potential inhibitors. Once activated, TOPK interacts with and phosphorylates a wide variety of proteins serving as mitotic regulators, oncogenes, or tumor suppressors. Several common TOPK inhibitors and many potential compounds targeting TOPK are developed and studied in preclinical evaluation.

TOPK, as a serine–threonine kinase, belongs to the MAPKK family and is closely associated with several biological activities. Previous studies have reported that TOPK directly phosphorylates ERKs, histone H3 (Ser10), histone H2AX (Ser139), peroxiredoxin (Ser32), JNK (Thr183/Tyr185), and PRPK (Ser250), indicating that it is a promising target in cancer related to growth, DNA damage, the cell cycle, and apoptosis. As shown in this review, various research articles confirm the function of TOPK both as an attractive target for drug discovery and a valuable cancer biomarker, although some features of TOPK have yet to be revealed. Therefore, we summarized the known proteins that regulate TOPK or are regulated by TOPK; additionally, we provided a current list of TOPK inhibitors and compounds that have been shown to modulate the TOPK pathway (Figure 3). Furthermore, we also introduced the biological characteristics related to the effects of targeting TOPK; despite this, the degree to which existing inhibitors are toxic, i.e., induce blood toxicity [23], is yet to be addressed.

The generation of toxic responses determines the direction of drug development to some extent. Important questions must therefore be carefully considered: How can a lack of solubility and increased blood toxicity be avoided in drugs? How can the sensitivity of cancer cells to drugs be improved? How can specificity be designed to target a drug?

At present, the distraction of mitogenic activating factors and the manipulation of cell cycle regulatory proteins are effective strategies to suppress cancer cell proliferation. Although the mitogenic activators (MEK inhibitor) and CDKs (CDK4/6 inhibitors) play important roles in cell division and regulate transcription, both of them produced the side effect of systemic toxicity or acquired resistance [123] and reduction of neutropenia [124], respectively. Nevertheless, our GO analysis revealed that TOPK is involved in the inhibition of mitotic activators, manipulation of cell cycle regulatory proteins or signal transduction, and regulation phosphorylation, which all promote the idea of therapeutic strategies and drug development to disturb tumor proliferative. Therefore, TOPK serves as a novel alternative to the current cell cycle regulatory targets. Recently, researchers have turned their attention to natural compounds, which play a significant role in antitumor activity because they not only possess multiple targets but also have low toxicity and few adverse effects. Furthermore, researchers can take advantage of high-resolution 3D protein structures and computer-docking tools to identify natural compounds or FDA-approved drugs that target TOPK or other kinases; this can improve the precision towards targets and overcome the side effects of compounds such as glycyrol and pantoprazole [21,110].

MicroRNAs (miRNAs/miRs) are noncoding RNAs that suppress gene expression by inhibiting the translation of target mRNAs or by degrading them. miRNAs can interact directly with the 3’-untranslated region of target mRNAs and lead to inhibition of proliferation, differentiation, metastasis, and apoptosis [125]. For instance, miR-216b and miR-770-5p have been demonstrated to inhibit proliferation of lung adenocarcinoma and breast cancer by targeting TOPK [57,112]. In addition, evidence demonstrates that patients with KRAS, BRAF, or PTEN mutations experience fewer clinical responses to drugs such as cetuximab and panitumumab. Moreover, overexpression of TOPK is significantly related to KRAS and BRAF mutations. Therefore, targeting TOPK appears to be valuable for overcoming mutations of KRAS or BRAF, as well as for patients who are resistant to EGFR drugs.

In addition, the molecular mechanisms involved in TOPK activation and the way it induces cancer are perplexing. Previous studies have reported that MAPKs can self-activate (auto-phosphorylation) under specific conditions, indicating that they are capable of such activity and that it is tightly regulated [126,127]. However, the activity of ERKs as well as the other MAPKs is elevated upon stimulation by phosphorylation of two residues in the activation loop. This activating phosphorylation is usually induced by MAPKKs (MEK1/2 in the case of ERK) but can also be achieved by autophosphorylation. Indeed, previous studies have shown that the autophosphorylation and autoactivation of p38 mitogen-activated protein kinase promoted by TAB1 abolishes its cardiac toxicity [127]. In mitogen-activated protein kinases (MAPKs), autophosphorylation regulation is very tight and occurs via unique mechanisms and structural motifs specific to each MAPK. Although TOPK belongs to the MAPK family, there are currently no reports indicating that it is capable of autophosphorylation. Therefore, additional investigation exploring the downstream protein mechanism of TOPK autophosphorylation on the MAPK pathway is warranted. 

Li et.al reported that TOPK negatively regulates p38α activity by enhancing the stability of the p38α-specific phosphatase MKP1 [3]. This finding suggests that TOPK may inhibit p38α downstream signaling. However, to date, there have been no reports indicating that TOPK phosphorylates both Tyr and Thr residues in the activation loop of all MAPKs. Furthermore, ERK and p38 have been shown to be activated downstream of TOPK; however, other studies have shown that ERK is involved in regulating TOPK activity [10]. This feedback loop suggests that inhibitors of ERK or p38 could be combined for the treatment of cancers with TOPK-induced proliferation. All of the above motivates us to study the mechanism of TOPK in combined treatment.

Currently, the functions of TOPK and its inhibitors have been reviewed in individual studies [128]; however, no comprehensive or intuitive works have been published, and, thus, a strength of the current paper is that it is more profound and broader than previous reports. In addition, drugs targeting TOPK are not used as frequently as AKT or AURKA drugs in clinical settings [129,130]; however, TOPK is identified here as a novel prognostic predictive factor and is involved in several signaling pathways, such as the MAPK and AKT pathways. Thus, it can be considered indispensable for future studies. With an increasing number of natural compounds detected for targeting TOPK, the implication is that TOPK inhibitors will eventually be used in clinical settings. 

## Figures and Tables

**Table 1:** Proteins regulated by TOPK involved in various pathways.

Name	Effect Site	Function	Mechanisms	Ref
AKT	Phosphorylation of Ser473	TOPK promotes AKT phosphorylation at Ser473 and decreases PTEN levels	PTEN–AKT pathway	[9]
AR	N/A	promotes proliferation via upregulation of PBK	A reciprocal feedback between TOPK and AR	[83]
CDK1	Binds to #234–275 of TOPK, the C-terminal side of the PRC1-binding sequence	Involved in mitosis	Cell cycle arrest	[4]
CHK1	CHFR ubiquitinates and regulates TOPK	Involved in mitosis	S phase arrest	[53]
CHFR	TOPK interacts with CHK1	Involved in mitosis	G2 to M progression	[49]
c-MYC	c-Myc activate TOPK	Represses TOPK transcription	Inhibition of c-Myc- E2F1-PBK signaling that decreases cell growth and survival	[84]
CCNB2	N/A	Represses TOPK transcription	PBK is essential for radioresistance by transcriptional regulation of CCNB2	[56]
C2H2	Phosphorylate C2H2 linker sequences	Regulate mitosis	Cell cycle arrest	[85]
CREB/ATF	Binding the PBK promoter (−312 bp)	Represses TOPK transcription	Associates with TOPK promoter	[86]
ERK2	TOPK–ERK interaction	Increases ERK activity	MAPK–ERK pathway	[10]
E2F1	Binding sites within the PBK promoter (−146 bp)	Represses TOPK transcription	Associates with TOPK promoter	[86]
FoxM1	PBK promoter	Increases the stability and activity of TOPK	FoxM1/PBK/β-catenin signaling	[40]
HIF1A	N/A	Transcription of HIF1A increased by TOPK regulation	HIF-1a/snail pathway	[87]
H3F3A	Phosphorylation of S10	Involved in mitosis	Cell cycle arrest	[88]
hDlg	Binds to the PDZ2 domain of hDlg	PBK and hDlg are phosphorylated at mitosis; phosphorylation of PBK is required for its kinase activity	PBK could link hDlg or other PDZ-containing proteins to signal transduction pathways regulating the cell cycle or cellular proliferation	[88]
IL-6	N/A	Increases TOPK activity	Cell cycle arrest	[89]
JNK	Binds to the JNK	TOPK regulates UVB-induced JNK1 activity	TOPK enhanced the ability of JNKs to mediate H-Ras-induced cell transformation	[19]
LGN/GPSM2	Thr450	Mitosis activity	Cell cycle arrest	[55]
MAPK11	TOPK-P38 interaction	Mitosis activity	p38MAPK activity and regulation of the DNA damage response	[44]
MET	Phosphorylates Y74 of TOPK	Increases the activity of TOPK	COX2/MET/TOPK signaling pathway	[81]
MKP1	Phosphorylation of Ser-359	TOPK directly binds with and phosphorylates MKP1	p38 signaling pathway	[3]
NF-κB	N/A	Represses TOPK transcription	ERK pathway	[22]
NF-κB	N/A	Activated by TOPK	Upregulation of snail/slug in TGF-β1 signaling	[90]
PRDX1	Ser32	TOPK interacts with and phosphorylates Prx1	Induced apoptosis	[62]
PRC1	T481	TOPK interacts with and phosphorylates PRC1	Stopping formation of mitotic spindles and spindle midzone	[4]
RORγ	N/A	PBK interacts with RORγ and modulates RORγ protein stability	AR-PBK- RORγ-AR pathway	[91]
SRC	Phosphorylation of TOPK at Y74 and Y272	Increases the stability and activity of TOPK	TOPK–histone H3 pathway	[42]
TP53	TOPK interacts with p53 through a DNA-binding domain	PBK modulation of p21 expression is dependent on p53	Cell cycle arrest	[18]
TBX3	TBX3 was activated by TOPK	TOPK upregulates T-box transcription factor TBX3	TGF-b1/Smad signaling	[20]
ULK1	Ser469, Ser495, and Ser533	TOPK binds and phosphorylates ULK1	Promoting ubiquitination degradation of ULK1	[70]
YAP1	N/A	Represses TOPK transcription	Hippo-YAP/TAZ pathway	[92]
γ-catenin	N/A	TOPK binds with γ-catenin	Src/GSK3β/STAT3 pathway	[12]

**Table 2:** Confirmed targets and the efficacy of inhibitors and compounds in vitro and in vivo.

Compound Name	Cancer	In Vitro	Concentration	In Vivo	Dose	Efficacy	Ref
HI-TOPK-32	Colon cancer (HCT-116)	Kinase assay	0–5 μmol/L	Patient-derived xenograft	1 or 10 mg/kg	More than 60% relative to the vehicle-treated group	[22]
	Skin cancer SKH1 (Crl:SKH1-Hr^hr^) hairless mice	Kinase assay	0–5 μM	37-kJ/m^2^ UVA and 1.8-kJ/m^2^ UVB to 60-kJ/m^2^ UVA and 2.9-kJ/m^2^ UVB	0.1% or 1% (*w*/*w*) of HI-TOPK-032	Epidermal thickness decreased	[93]
	Nasopharyngeal carcinoma		0–2 μM	Cell-derived xenograft (CNE-2)	5 mg/kg	Significantly decreased tumor volume	[78]
	Adult T-cell leukemia/lymphoma			Cell-derived xenograft (HUT-102)	12.5 mg/kg	Inhibited tumor growth	[17]
	Glioblastoma multiforme		0–10 μM	Cell-derived xenograft	5 and 10 mg/kg	Significantly inhibited	[94]
	Adrenocortical carcinoma (CU-ACC1)		0–4 μM	Cell-derived xenograft	10 mg/kg	Significant decreases	[100]
OTS514	Lung cancer			Human LU-99 lung cancer	Liposomal OTS514 (1, 2.5, and 5 mg/kg)	2.5 and 5 mg/kg of liposomal formulation of OTS514 resulted in TGIs of 90.3	[23]
OTS514	Ovarian cancer			Human ES-2 cells and patient-derived ovarian cancer cells	25 or 50 mg/kg, 10 and 100 nmol/L, of OTS514	No tumor growth and strong growth-inhibitory effects	[24]
	Anti-myeloma (HMCL H929)		0–100 nmol/L	Cell-derived ovarian cancer cells	50 or 100 mg/kg	Significant inhibition	[101]
	Prostate cancer		0–100 nM				[102]
	Kidney cancer		VMRC-RCW (IC50: 19.9 nM) Caki1 (IC50: 27.8 nM), Caki2 (IC50: 20.1 nM), 769P (IC50: 20.7 nM) 786O (IC50: 44.1 nM)				
	GBM		0–10 μM	Cell-derived xenograft (U87 and U251)	Tail vein injection of OTS514 (20 mg/kg/day)	Significantly inhibited	[56]
OTS964	Lung cancer			Human LU-99 lung cancer	Liposomal OTS964 (40 mg/kg)		[23]
	Glioblastoma	Clonal assay	0–600 nM				[96]
[18F]FE-OTS964	Glioblastoma (U87 cell)			Cell-derived ovarian cancer cells		Significant inhibition	[35]
ADA-07	Skin cancer	Kinase assay	0–5 μM	SUV irradiation source (36-kJ/m^2^ UVA and 1.8-kJ/m^2^ UVB to 60-kJ/m^2^ UVA and 2.9-kJ/m^2^ UVB)	0.1 or 1 mg of ADA-07	Significantly reduced tumor volume and multiplicity	[61]
Ilaprazole	Colorectal cancer Lung cancer Ovary cancer Pancreas cancer	Virtual ligand screening		Human HCT116 xenograft model	75 and 150 mg/kg	Significant decreases in tumor volume	[97]
Pantoprazole	Colon cancer	Kinase assay		Human HCT-116 cancer cell xenograft model	100 mg/kg	Significantly inhibited	[21]
Atorvastatin	ER-negative breast cancer cells		10 μM				[92]
1-phenyl phenanthridin-6(5H)-1	Colon cancer HCT-116/HCT-15/SW480	TOPK kinase		Human cancer cell Pharmacokinetic assay in rats	10 mg/kg, 15 mg/kg IV 2 mg/kg (iv) and 15 mg/kg (po)	Suppressed tumor growth with TGI >79.7% Quantified compound concentration	[103]
3’deoxysappanchalcone	Colon cancer	Kinase assay	0–20 μM				[2]
	Skin cancer	Kinase assay	0–20 μM	SK-MEL-2 cell xenograft model	10 and 20 mg/kg	inhibited	[104]
Acetylshikonin	Colon cancer	Kinase assay	0–10 μM	Patient-derived xenograft	120 or 160 mg/kg	Significantly reduced	[1]
Baicalin	Lung cancer	kinase assay	0–100 μM	Human H441 cancer cell xenograft model	20 or 50 mg/kg administered by i.p.	50-mg/kg baicalin: grew remarkably more slowly	[105]
Cefradine	Skin inflammation	Kinase assay	0–2 mM	100-kJ/m^2^ SUV irradiation	Smeared with cefradine (100 mg/kg)	Thickness of epidermis in mice treated with 100 mg/kg of cefradine remarkably reduced	[60]
Coffee phenolic phytochemicals	Colon cancer	Kinase assay	0–80 μM	CT-26	0.5, 1.0, and 2.0 g/kg (iv)	Metastasis inhibited	[106]
Cyanidin-3-O-glucoside	Colorectal cancer	Kinase assay					[107]
Eupafolin	Esophagus cancer	Kinase assay	0–100 μM	Patient-derived xenograft	20 or 50 mg/kg	Inhibits	[41]
Ginsenoside Rh2	Colon cancer	Kinase assay	0–50 μM	Human HCT-116 xenograft model	10 or 50 mg/kg	suppressed tumor growth by 49% and 78% relative to the vehicle	[108]
Gossypetin	Cutaneous basal cell carcinoma	Kinase assay		SKH-1 hairless mice		Significant reduction	[109]
Glycyrol	Lung cancer (A549, HCC827, HCC827GR, PC9, and PC9GR10)	Kinase assay	0–10 μM	Human A549 cancer cell xenograft mouse model	20 mg/kg (i.p.)	Significantly inhibited	[110]
Glycycoumarin	Liver cancer	Kinase assay	0–75 μM	Human cancer cell HepG2 xenograft mouse model	20 mg/kg (i.p.)	Significant suppression of tumor growth	[111]
MicroRNA-216b-3p	Lung adenocarcinoma cell (A549, GLC-82, and H358) Colorectal cancer lines (DLD1, HCT-116, SW480, COLO-678, HT29, etc.)						[59,60]
miR-770-5p	MCF7, A549, and HCT-116 cells		0–100 nM	Human cell cancer HCT-116 xenograft mouse model	100 nM (i.p.) and IR + 10 nM (i.p.)	Suppresses PBK expression, resulting in tumor growth retardation	[112]
Paeonol	Skin inflammation	MST assay	0–100 μM	100-kJ/m^2^ SUV irradiation	Smeared on skin with 60 mg/kg Paeonol before irradiation	60 mg/kg inhibits inflammation	[113]
SKLB-C05	Colorectal cancer	Kinase assay and thermal shift assay	0–400 nM	Human cancer cells (HCT-116, SW480, and DLD1) in a xenograft mouse model	10 and 20 mg/kg of SKLB-C05	TGI values of 64.3% and 92.4% in HCT116, 54.2% and 88.9% in SW480, and 46.3% and 67.5% in DLD-1	[48]
Scutellarin	Melanoma cell	Kinase assay					[114]
Sulfasalazine	Thyroid cancer cell (K1, SW579, FTC133, and TT)	Kinase assay and microscale thermophoresis	0–150 μM				[98]

## Data Availability

All data was generated in this review are available from Gene Expression Profiling Interactive Analysis (http://gepia.cancer-pku.cn/, (accessed on 25 March 2021) http://ualcan.path.uab.edu/ (accessed on 25 March 2021) and https://string-db.org/ (accessed on 25 March 2021)).

## Abbreviations

ACC: adrenocortical carcinoma; BLCA: bladder urothelial carcinoma; BRCA: breast invasive carcinoma; CESC: cervical squamous cell carcinoma; CHOL: cholangiocarcinoma; COAD: colon adenocarcinoma; DLBC: diffuse large B-cell lymphoma; ESCA: esophageal carcinoma; GBM: glioblastoma multiforme; HNSC: head and neck squamous cell carcinoma; KICH: kidney chromophobe carcinoma; KIRC: kidney renal clear cell carcinoma; KIRP: kidney renal papillary cell carcinoma; LGG: brain lower grade glioma; OV: ovarian serous cystadenocarcinoma; MESO: mesothelioma; LIHC: liver hepatocellular carcinoma; LUAD: lung adenocarcinoma; LUSC: lung squamous cell carcinoma; PAAD: pancreatic adenocarcinoma; PCPG: pheochromocytoma and paraganglioma; PRAD: prostate adenocarcinoma; READ: rectum adenocarcinoma; SARC: sarcoma; SKCM: skin cutaneous melanoma; LAML: acute myeloid leukemia; TGCT: testicular germ cell tumors; THCA: thyroid carcinoma; THYM: thymoma; STAD: stomach adenocarcinoma; UCEC: uterine corpus endometrial carcinoma; UCS: uterine carcinosarcoma; UVM: uveal melanoma; AKT: V-akt murine thymoma viral oncogene homolog; AURKA: Aurora kinase A; TOPK: T-lymphokine-activated killer cell-originated protein kinase; KRAS: kirsten rat sarcoma viral oncogene; BRAF: v-raf murine sarcoma viral oncogene homolog B1; JNK: c-Jun N-terminal kinase; MEK: mitogen-activated proteinkinase kinase 1; ERK: Extracellular regulated protein kinases; MAPK: mitogen-activated protein kinase; NOTCH1: Neurogenic Locus Notch Homolog Protein 1; PI3K: Phosphatidylinositol-3 kinase; PTEN: phosphatase and tensin homologue deleted from chromosome 10; KEGG: Kyoto Encyclopedia of Genes and Genomes; GO: Gene Ontology. MF: molecular function; BP: biological process, CC: cellular component.
